# 1-(2-Chloro­benzo­yl)-3-(3-meth­oxy­phen­yl)thio­urea

**DOI:** 10.1107/S1600536812048830

**Published:** 2012-12-05

**Authors:** M. Khawar Rauf, Masahiro Ebihara, Amin Badshah

**Affiliations:** a Department of Chemistry, Quaid-i-Azam University, Islamabad 45320, Pakistan; b Department of Chemistry, Faculty of Engineering, Gifu University Yanagido, Gifu 501-1193, Japan

## Abstract

The title compound, C_15_H_13_ClN_2_O_2_S, exists in the solid state in its thione form with typical thio­urea C—S and C—O bonds lengths as well as shortened C—N bonds. An intra­molecular N—H⋯O hydrogen bond stabilizes the mol­ecular conformation. In the crystal, N—H⋯S hydrogen bonds link the mol­ecules into centrosymmetric dimers.

## Related literature

For previous work on *N*,*N′*-disubstituted thio­ureas, see: Rauf *et al.* (2012 ▶). For a description of the Cambridge Structural Database, see: Allen (2002 ▶).
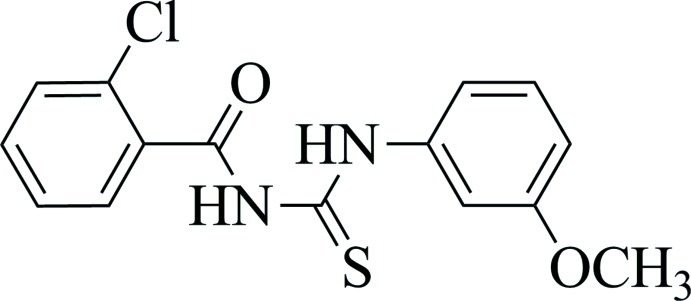


## Experimental

### 

#### Crystal data

C_15_H_13_ClN_2_O_2_S
*M* *_r_* = 320.78
Triclinic, 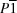

*a* = 6.276 (3) Å
*b* = 10.202 (5) Å
*c* = 11.411 (5) Å
α = 94.541 (7)°
β = 93.305 (6)°
γ = 96.918 (7)°
*V* = 721.3 (6) Å^3^
*Z* = 2
Mo *K*α radiation
μ = 0.41 mm^−1^
*T* = 123 K
0.45 × 0.36 × 0.20 mm

#### Data collection

Rigaku/MSC Mercury CCD diffractometer
5698 measured reflections
3222 independent reflections
3071 reflections with *I* > 2σ(*I*)
*R*_int_ = 0.052

#### Refinement

*R*[*F* ^2^ > 2σ(*F* ^2^)] = 0.035
*wR*(*F* ^2^) = 0.088
*S* = 1.09
3222 reflections
191 parameters
H-atom parameters constrained
Δρ_max_ = 0.33 e Å^−3^
Δρ_min_ = −0.34 e Å^−3^

### 

Data collection: *CrystalClear* (Molecular Structure Corporation & Rigaku, 2001 ▶); cell refinement: *CrystalClear*; data reduction: *CrystalClear*; program(s) used to solve structure: *SIR97* (Altomare *et al.*, 1999 ▶); program(s) used to refine structure: *SHELXL97* (Sheldrick, 2008 ▶); molecular graphics: *ORTEPII* (Johnson, 1976 ▶); software used to prepare material for publication: *Yadokari-XG* (Wakita, 2001 ▶; Kabuto *et al.*, 2009 ▶).

## Supplementary Material

Crystal structure: contains datablock(s) I, global. DOI: 10.1107/S1600536812048830/mw2100sup1.cif

Structure factors: contains datablock(s) I. DOI: 10.1107/S1600536812048830/mw2100Isup2.hkl

Supplementary material file. DOI: 10.1107/S1600536812048830/mw2100Isup3.cml

Additional supplementary materials:  crystallographic information; 3D view; checkCIF report

## Figures and Tables

**Table 1:** Hydrogen-bond geometry (Å, °)

*D*—H⋯*A*	*D*—H	H⋯*A*	*D*⋯*A*	*D*—H⋯*A*
N2—H2⋯O1	0.88	1.95	2.6500 (17)	135
N1—H1⋯S1^i^	0.88	2.64	3.4080 (17)	146

Symmetry code: (i) 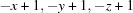.

**Fig. 1.**
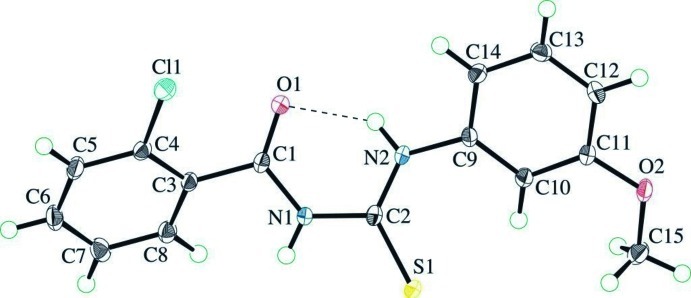
Molecular structure of (I) showing atom numbering scheme. Displacement ellipsoids are drawn at the 50% probability level. Hydrogen bonds are shown as dashed lines.

**Fig. 2.**
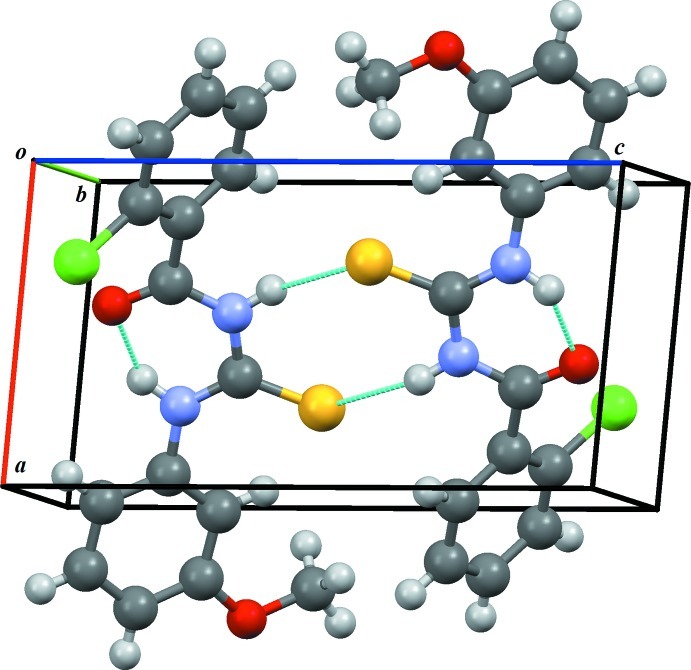
Packing diagram of (I) with view onto the ab plane. Hydrogen bonds shown as dashed lines.

## supplementary crystallographic information

### Comment

The background to this study has been set out in our previous work on the structural and coordination chemistry of *N*,*N'*-disubstituted thioureas (Rauf *et al.*, 2012). Herein, as a continuation of these studies, the structure of the title compound (I) is described. A depiction of the molecule is given in Fig. 1. Bond lengths and angles are comparable to those for other *N*,*N'*-disubstituted thioureas reported in the Cambridge Structural Database (Allen, 2002). The molecule exists in the thione form with typical thiourea C—S and C—O bonds as well as shortened C—N bond lengths. The molecule features an intramolecular N—H···O hydrogen bond and in the solid molecules associate *via* intermolecular N—H···S hydrogen bonds which link the molecules into centrosymmetric dimers (Table 1 and Fig. 2).

### Experimental

Freshly prepared 2-chlorobenzoyl isothiocyanate (1.98 g, 10 mmol) was stirred in acetone (50 mL) for 30 minutes. Distilled 3-methoxyaniline (1.23 g, 10 mmol)was then added and the resulting mixture was stirred for 1 h. The reaction mixture was then poured into acidified (pH 4) water and stirred. The solid product was separated and washed with deionized water and purified by recrystallization from methanol/dichloromethane (1:10 *v/v*) to give fine crystals of (I) with an overall yield of 93% (2.98 g). M.P; 109–109.5°C. Anal. calcd. for C_15_H_13_ClN_2_O_2_S; C, 56.16 H, 4.08 N, 8.73 S, 10.00 Found: C, 56.12 H, 4.07 N, 8.73 S, 9.98.

### Refinement

Hydrogen atoms were included in calculated positions and refined as riding on their parent atom with N—H = 0.88 Å and *U*_iso_(H) = 1.2U(N_eq_), C_aromatic_—H = 0.95 Å and *U*_iso_(H) = 1.2U(C_eq_) or C—H = 0.98 Å and *U*_iso_(H) = 1.5U(C_eq_), for methyl C atoms.

### Crystal data

**Table:**

C_15_H_13_ClN_2_O_2_S	*Z* = 2
*M**_r_* = 320.78	*F*(000) = 332
Triclinic, *P*1	*D*_x_ = 1.477 Mg m^−^^3^
Hall symbol: -P 1	Mo *K*α radiation, λ = 0.71070 Å
*a* = 6.276 (3) Å	Cell parameters from 2546 reflections
*b* = 10.202 (5) Å	θ = 3.3–27.5°
*c* = 11.411 (5) Å	µ = 0.41 mm^−^^1^
α = 94.541 (7)°	*T* = 123 K
β = 93.305 (6)°	Block, colorless
γ = 96.918 (7)°	0.45 × 0.36 × 0.20 mm
*V* = 721.3 (6) Å^3^	

### Data collection

**Table:**

Rigaku/MSC Mercury CCD diffractometer	3071 reflections with *I* > 2σ(*I*)
Radiation source: Rotating Anode	*R*_int_ = 0.052
Graphite Monochromator monochromator	θ_max_ = 27.5°, θ_min_ = 3.3°
Detector resolution: 14.62 pixels mm^-1^	*h* = −8→5
ω scans	*k* = −13→13
5698 measured reflections	*l* = −14→14
3222 independent reflections	

### Refinement

**Table:**

Refinement on *F*^2^	Primary atom site location: structure-invariant direct methods
Least-squares matrix: full	Secondary atom site location: difference Fourier map
*R*[*F*^2^ > 2σ(*F*^2^)] = 0.035	Hydrogen site location: inferred from neighbouring sites
*wR*(*F*^2^) = 0.088	H-atom parameters constrained
*S* = 1.09	*w* = 1/[σ^2^(*F*_o_^2^) + (0.0309*P*)^2^ + 0.5054*P*] where *P* = (*F*_o_^2^ + 2*F*_c_^2^)/3
3222 reflections	(Δ/σ)_max_ = 0.001
191 parameters	Δρ_max_ = 0.33 e Å^−^^3^
0 restraints	Δρ_min_ = −0.34 e Å^−^^3^

### Special details

**Table:**

Geometry. All e.s.d.'s (except the e.s.d. in the dihedral angle between two l.s. planes) are estimated using the full covariance matrix. The cell e.s.d.'s are taken into account individually in the estimation of e.s.d.'s in distances, angles and torsion angles; correlations between e.s.d.'s in cell parameters are only used when they are defined by crystal symmetry. An approximate (isotropic) treatment of cell e.s.d.'s is used for estimating e.s.d.'s involving l.s. planes.
Refinement. Refinement of *F*^2^ against ALL reflections. The weighted *R*-factor *wR* and goodness of fit *S* are based on *F*^2^, conventional *R*-factors *R* are based on *F*, with *F* set to zero for negative *F*^2^. The threshold expression of *F*^2^ > σ(*F*^2^) is used only for calculating *R*-factors(gt) *etc*. and is not relevant to the choice of reflections for refinement. *R*-factors based on *F*^2^ are statistically about twice as large as those based on *F*, and *R*- factors based on ALL data will be even larger.

### Fractional atomic coordinates and isotropic or equivalent isotropic displacement parameters (Å^2^)

**Table:**

	*x*	*y*	*z*	*U*_iso_*/*U*_eq_	
C1	0.6542 (2)	0.57142 (13)	0.79707 (12)	0.0144 (3)	
O1	0.58303 (17)	0.56062 (10)	0.89370 (9)	0.0186 (2)	
N1	0.56572 (19)	0.49567 (12)	0.69582 (11)	0.0155 (2)	
H1	0.6364	0.5052	0.6320	0.019*	
C2	0.3774 (2)	0.40595 (13)	0.68314 (13)	0.0147 (3)	
S1	0.28505 (7)	0.34880 (4)	0.54559 (3)	0.02392 (12)	
N2	0.28727 (18)	0.37856 (12)	0.78283 (10)	0.0148 (2)	
H2	0.3660	0.4085	0.8479	0.018*	
C3	0.8470 (2)	0.66726 (14)	0.77733 (12)	0.0149 (3)	
C4	0.8823 (2)	0.79519 (14)	0.83489 (13)	0.0168 (3)	
C5	1.0642 (3)	0.88129 (15)	0.81696 (14)	0.0217 (3)	
H5	1.0863	0.9678	0.8566	0.026*	
C6	1.2135 (2)	0.84109 (16)	0.74122 (15)	0.0236 (3)	
H6	1.3379	0.9001	0.7292	0.028*	
C7	1.1816 (2)	0.71488 (16)	0.68298 (14)	0.0219 (3)	
H7	1.2837	0.6875	0.6309	0.026*	
C8	0.9996 (2)	0.62846 (15)	0.70116 (13)	0.0179 (3)	
H8	0.9787	0.5420	0.6614	0.021*	
Cl1	0.69817 (6)	0.85411 (4)	0.92737 (3)	0.02243 (11)	
C9	0.0829 (2)	0.30814 (13)	0.79995 (12)	0.0141 (3)	
C10	−0.0238 (2)	0.20764 (14)	0.72013 (13)	0.0166 (3)	
H10	0.0399	0.1801	0.6503	0.020*	
C11	−0.2262 (2)	0.14852 (14)	0.74534 (13)	0.0164 (3)	
C12	−0.3209 (2)	0.18776 (14)	0.84774 (13)	0.0181 (3)	
H12	−0.4605	0.1482	0.8627	0.022*	
C13	−0.2092 (2)	0.28502 (15)	0.92748 (13)	0.0186 (3)	
H13	−0.2712	0.3107	0.9984	0.022*	
C14	−0.0068 (2)	0.34540 (14)	0.90434 (13)	0.0169 (3)	
H14	0.0697	0.4116	0.9595	0.020*	
O2	−0.34498 (17)	0.04884 (11)	0.67328 (10)	0.0236 (3)	
C15	−0.2488 (3)	0.00094 (16)	0.57046 (14)	0.0243 (3)	
H15C	−0.2194	0.0732	0.5197	0.037*	
H15A	−0.3473	−0.0710	0.5274	0.037*	
H15B	−0.1138	−0.0322	0.5937	0.037*	

### Atomic displacement parameters (Å^2^)

**Table:**

	*U*^11^	*U*^22^	*U*^33^	*U*^12^	*U*^13^	*U*^23^
C1	0.0126 (6)	0.0132 (6)	0.0166 (7)	−0.0001 (5)	−0.0020 (5)	0.0008 (5)
O1	0.0193 (5)	0.0203 (5)	0.0144 (5)	−0.0045 (4)	0.0003 (4)	0.0006 (4)
N1	0.0148 (6)	0.0161 (6)	0.0139 (6)	−0.0037 (4)	0.0018 (4)	−0.0010 (4)
C2	0.0147 (6)	0.0110 (6)	0.0176 (7)	−0.0007 (5)	0.0001 (5)	−0.0003 (5)
S1	0.0298 (2)	0.0236 (2)	0.01342 (19)	−0.01405 (15)	0.00077 (14)	−0.00207 (14)
N2	0.0127 (5)	0.0153 (6)	0.0147 (6)	−0.0028 (4)	−0.0013 (4)	−0.0005 (4)
C3	0.0137 (6)	0.0156 (6)	0.0141 (6)	−0.0021 (5)	−0.0029 (5)	0.0029 (5)
C4	0.0175 (7)	0.0171 (7)	0.0147 (6)	−0.0009 (5)	−0.0030 (5)	0.0017 (5)
C5	0.0230 (7)	0.0183 (7)	0.0207 (7)	−0.0069 (6)	−0.0058 (6)	0.0030 (6)
C6	0.0161 (7)	0.0268 (8)	0.0260 (8)	−0.0070 (6)	−0.0048 (6)	0.0099 (6)
C7	0.0136 (7)	0.0283 (8)	0.0239 (8)	0.0003 (6)	0.0003 (5)	0.0071 (6)
C8	0.0148 (6)	0.0184 (7)	0.0197 (7)	0.0001 (5)	−0.0022 (5)	0.0026 (5)
Cl1	0.0263 (2)	0.01867 (18)	0.02099 (19)	0.00013 (14)	0.00284 (14)	−0.00308 (13)
C9	0.0124 (6)	0.0128 (6)	0.0164 (7)	−0.0008 (5)	−0.0007 (5)	0.0031 (5)
C10	0.0163 (7)	0.0162 (7)	0.0163 (7)	−0.0011 (5)	0.0027 (5)	−0.0015 (5)
C11	0.0152 (6)	0.0144 (6)	0.0183 (7)	−0.0021 (5)	−0.0003 (5)	−0.0002 (5)
C12	0.0144 (6)	0.0178 (7)	0.0220 (7)	−0.0003 (5)	0.0042 (5)	0.0033 (5)
C13	0.0204 (7)	0.0188 (7)	0.0168 (7)	0.0020 (5)	0.0047 (5)	0.0009 (5)
C14	0.0186 (7)	0.0150 (6)	0.0159 (7)	−0.0002 (5)	−0.0004 (5)	−0.0005 (5)
O2	0.0190 (5)	0.0236 (6)	0.0239 (6)	−0.0095 (4)	0.0047 (4)	−0.0077 (4)
C15	0.0230 (7)	0.0252 (8)	0.0220 (8)	−0.0020 (6)	0.0012 (6)	−0.0074 (6)

### Geometric parameters (Å, º)

**Table:**

C1—O1	1.2213 (18)	C7—H7	0.9500
C1—N1	1.3862 (18)	C8—H8	0.9500
C1—C3	1.5007 (19)	C9—C14	1.392 (2)
N1—C2	1.3981 (18)	C9—C10	1.3948 (19)
N1—H1	0.8800	C10—C11	1.394 (2)
C2—N2	1.3334 (19)	C10—H10	0.9500
C2—S1	1.6758 (16)	C11—O2	1.3713 (17)
N2—C9	1.4243 (18)	C11—C12	1.394 (2)
N2—H2	0.8800	C12—C13	1.385 (2)
C3—C4	1.401 (2)	C12—H12	0.9500
C3—C8	1.401 (2)	C13—C14	1.393 (2)
C4—C5	1.389 (2)	C13—H13	0.9500
C4—Cl1	1.7370 (16)	C14—H14	0.9500
C5—C6	1.387 (2)	O2—C15	1.4288 (19)
C5—H5	0.9500	C15—H15C	0.9800
C6—C7	1.388 (2)	C15—H15A	0.9800
C6—H6	0.9500	C15—H15B	0.9800
C7—C8	1.392 (2)		
			
O1—C1—N1	123.20 (13)	C7—C8—H8	119.6
O1—C1—C3	122.98 (12)	C3—C8—H8	119.6
N1—C1—C3	113.82 (12)	C14—C9—C10	120.87 (13)
C1—N1—C2	127.64 (12)	C14—C9—N2	115.32 (12)
C1—N1—H1	116.2	C10—C9—N2	123.81 (13)
C2—N1—H1	116.2	C11—C10—C9	118.42 (13)
N2—C2—N1	115.74 (12)	C11—C10—H10	120.8
N2—C2—S1	127.15 (11)	C9—C10—H10	120.8
N1—C2—S1	117.08 (11)	O2—C11—C12	115.39 (13)
C2—N2—C9	129.80 (12)	O2—C11—C10	123.32 (13)
C2—N2—H2	115.1	C12—C11—C10	121.30 (13)
C9—N2—H2	115.1	C13—C12—C11	119.29 (13)
C4—C3—C8	118.29 (13)	C13—C12—H12	120.4
C4—C3—C1	121.86 (13)	C11—C12—H12	120.4
C8—C3—C1	119.84 (13)	C12—C13—C14	120.44 (13)
C5—C4—C3	120.81 (14)	C12—C13—H13	119.8
C5—C4—Cl1	117.49 (12)	C14—C13—H13	119.8
C3—C4—Cl1	121.67 (11)	C9—C14—C13	119.61 (13)
C6—C5—C4	120.05 (14)	C9—C14—H14	120.2
C6—C5—H5	120.0	C13—C14—H14	120.2
C4—C5—H5	120.0	C11—O2—C15	117.13 (12)
C5—C6—C7	120.15 (14)	O2—C15—H15C	109.5
C5—C6—H6	119.9	O2—C15—H15A	109.5
C7—C6—H6	119.9	H15C—C15—H15A	109.5
C6—C7—C8	119.81 (15)	O2—C15—H15B	109.5
C6—C7—H7	120.1	H15C—C15—H15B	109.5
C8—C7—H7	120.1	H15A—C15—H15B	109.5
C7—C8—C3	120.88 (14)		
			
O1—C1—N1—C2	6.0 (2)	C6—C7—C8—C3	0.3 (2)
C3—C1—N1—C2	−174.52 (13)	C4—C3—C8—C7	−0.1 (2)
C1—N1—C2—N2	−8.8 (2)	C1—C3—C8—C7	−178.91 (13)
C1—N1—C2—S1	169.20 (12)	C2—N2—C9—C14	−151.59 (15)
N1—C2—N2—C9	169.91 (13)	C2—N2—C9—C10	28.9 (2)
S1—C2—N2—C9	−7.9 (2)	C14—C9—C10—C11	2.3 (2)
O1—C1—C3—C4	−39.5 (2)	N2—C9—C10—C11	−178.27 (13)
N1—C1—C3—C4	141.05 (14)	C9—C10—C11—O2	−179.88 (14)
O1—C1—C3—C8	139.31 (15)	C9—C10—C11—C12	−0.2 (2)
N1—C1—C3—C8	−40.16 (18)	O2—C11—C12—C13	177.99 (13)
C8—C3—C4—C5	−0.1 (2)	C10—C11—C12—C13	−1.7 (2)
C1—C3—C4—C5	178.72 (13)	C11—C12—C13—C14	1.6 (2)
C8—C3—C4—Cl1	177.95 (11)	C10—C9—C14—C13	−2.4 (2)
C1—C3—C4—Cl1	−3.24 (19)	N2—C9—C14—C13	178.10 (13)
C3—C4—C5—C6	0.1 (2)	C12—C13—C14—C9	0.4 (2)
Cl1—C4—C5—C6	−178.03 (12)	C12—C11—O2—C15	−176.82 (14)
C4—C5—C6—C7	0.1 (2)	C10—C11—O2—C15	2.9 (2)
C5—C6—C7—C8	−0.3 (2)		

### Hydrogen-bond geometry (Å, º)

**Table:**

*D*—H···*A*	*D*—H	H···*A*	*D*···*A*	*D*—H···*A*
N2—H2···O1	0.88	1.95	2.6500 (17)	135
N1—H1···S1^i^	0.88	2.64	3.4080 (17)	146

Symmetry code: (i) −*x*+1, −*y*+1, −*z*+1.
